# Glioblastoma Multiforme: A Look Inside Its Heterogeneous Nature

**DOI:** 10.3390/cancers6010226

**Published:** 2014-01-27

**Authors:** Maria-del-Mar Inda, Rudy Bonavia, Joan Seoane

**Affiliations:** 1 Translational Research Program, Vall d’Hebron Institute of Oncology (VHIO), Vall d’Hebron University Hospital, 119-129 Passeig Vall d’Hebron, Barcelona 08035, Spain; E-Mails: rbonavia@vhio.net (R.B.); jseoane@vhio.net (J.S.); 2 Catalan Institution of Research and Advanced Studies (ICREA), Barcelona 08035, Spain

**Keywords:** heterogeneity, glioblastoma, clonal evolution, cancer stem cell

## Abstract

Heterogeneity is a hallmark of tumors and has a crucial role in the outcome of the malignancy, because it not only confounds diagnosis, but also challenges the design of effective therapies. There are two types of heterogeneity: inter-tumor and intra-tumor heterogeneity. While inter-tumor heterogeneity has been studied widely, intra-tumor heterogeneity has been neglected even though numerous studies support this aspect of tumor pathobiology. The main reason has been the technical difficulties, but with new advances in single-cell technology, intra-tumor heterogeneity is becoming a key area in the study of cancer. Several models try to explain the origin and maintenance of intra-tumor heterogeneity, however, one prominent model compares cancer with a tree where the ubiquitous mutations compose the trunk and mutations present in subpopulations of cells are represented by the branches. In this review we will focus on the intra-tumor heterogeneity of glioblastoma multiforme (GBM), the most common brain tumor in adults that is characterized by a marked heterogeneity at the cellular and molecular levels. Better understanding of this heterogeneity will be essential to design effective therapies against this devastating disease to avoid tumor escape.

## 1. Introduction

No two individuals are alike. Even homozygous twins differ in some characteristics, these differences being caused by epigenetic and environmental factors. The same occurs in cancer, not only do tumors from different organs differ, but tumors located in the same organ are also different. This heterogeneity among tumors is what is known as inter-tumor heterogeneity. However, not only tumors with the same diagnosis differ, but also cells within a given tumor are different and this type of heterogeneity is known as intra-tumoral heterogeneity. The coexistence of those different clones within the same tumor is presumably caused by stochastic events, but their maintenance is under selective pressure and can be favored or disfavored by the interaction with other tumor clones or host cells.

The concept of tumor heterogeneity is absolutely not new. As early as in the nineteenth century, Rudolf Carl Virchow, considered one of the fathers of pathology, was the first to describe the pleomorphism of tumor cells [1]. This observation dates back much earlier than any knowledge about genetic alterations that are the drivers of tumor transformation. For decades, many observations based on immunohistochemical analysis have been made describing differences in morphology and protein expression between cells within a tumor sample. For many years, however, the concept was confined to intermingled presence of tumor and host cells, or simply random events within the tumor cell population. Differences were mostly attributed to infiltration of tumor cells in the surrounding tissue or vice versa and considered as the result of stochastic events. However, nowadays, there is the growing acceptance that heterogeneity is one of the key features of tumorigenesis responsible for tumor progression, resistance, metastatic potential and relapse. 

Intra-tumor heterogeneity confers an evolutionary advantage in the face of either microenvironmental fluctuations or selective pressure imposed by chemo- or radio-therapy. The pre-existence of clones resistant to determined therapies has been demonstrated in various types of tumors before the treatment [2,3]. These clones constitute the main cause of failure of targeted therapies and are responsible for tumor relapse after treatment.

These findings, together with the understanding that every tumor is different from any other, represent the main challenge in the design of new therapies. Thus, the study and understanding of tumor heterogeneity will represent an obligated path to the development of personalized therapies, in particular in the cases of tumors notoriously refractory to conventional therapies. In this review we will focus on glioblastoma multiforme (GBM), a type of tumor emblematic for inter- and intra-tumor heterogeneity and resistance to treatment. We will focus on intra-tumor heterogeneity, discussing different theories explaining its origin, as well as the methods employed for its study and its implications in diagnosis and therapy.

## 2. Origin of Heterogeneity in Cancer

**Figure 1:**
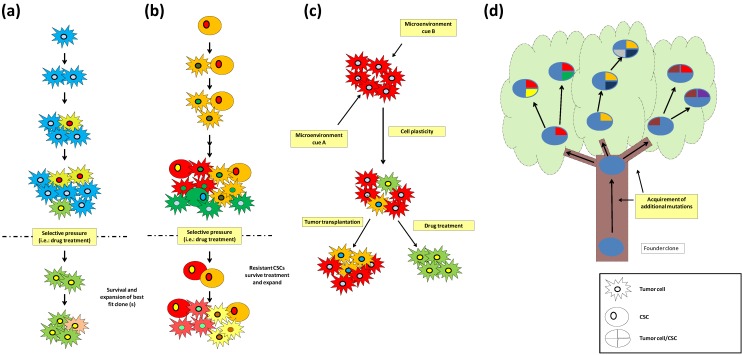
Models for the origin of intra-tumor heterogeneity. (**a**) Clonal evolution: tumor cells divide and acquire mutations, upon a selective pressure the most fit clone (s) will survive and cause tumor relapse; (**b**) CSC model: CSCs divide asymmetrically and generate CSCs and more differentiated tumor cells that can acquire mutations. Upon treatment, resistant CSCs will survive and cause tumor relapse; (**c**) Heterogeneity can be generated by cell plasticity in response to microenvironment cues that mainly through epigenetic mechanisms generate cells with different tumorigenic properties as in example tumor initiation capacity or drug resistance; (**d**) Branched tree model: Mutations shared by all tumor cells proceed from the founder clone which is depicted as the trunk of the tree. The branches are composed by tumor cells that acquire mutations present only in a subset of the tumor cells.

Early theories attempting to explain the ontogenesis of cancers were based on parallels of this process with the Darwinian theory of evolution. In the clonal evolution model (Figure 1a), genetic or epigenetic mutations appear randomly and any new phenotype is subjected to the pressure of natural selection. According to this model, best fit clone(s) will expand and outgrow the others, while heterogeneity would be explained as the presence of remaining weaker clones generated during tumor expansion. This variability would become important in the case of environmental changes, in particular those induced by chemo- or radiotherapy, when the previous acquisition of a resistant phenotype would allow a minor population to survive, expand, and become dominant [4].

More recently, the cancer stem cell (CSC) theory (Figure 1b) has become an accepted model of cancer initiation and evolution. This theory postulates a hierarchical organization in which a tumor generates from cells with stem cell characteristics, known as CSCs. By asymmetric division, these cells will maintain their population and, at the same time, generate more differentiated daughter cells with limited proliferation properties that constitute the bulk of the tumor, while CSCs will remain as a small subpopulation. In this view, heterogeneity is seen as the dualistic nature of CSCs (tumorigenic) and non-CSCs with various degrees of differentiation (non-tumorigenic), regardless of their genetic background [5] presumably determined by epigenetic changes [6]. However, this model is based in functional assays of tumor transplantation that might underestimate the tumorigenic potential of these cells depending on the mouse strain, the conditions of the assay, *etc*. [6,7]. Glioma CSCs were initially defined by the expression of the surface marker CD133 (prominin-1), and cells not expressing this marker were thought to lack tumorigenic potential [8]. More recently, it has been shown that a subpopulation of glioma cells characterized by high expression of CD44 and Id1 possess a stem-like phenotype [9]. Moreover, it is been demonstrated that these molecules can be used as CSC markers depending on the glioma subtype. While, CD133 seems to be expressed in proneural glioma CSCs, CD44 is highly expressed in mesenchymal glioma CSCs [10]. Other markers are under study, and supporting the fact that some CD133 negative cells are able to form tumors in immunocompromised mice [11], suggesting that additional markers are necessary for the identification of glioma CSCs due to their heterogeneous nature. Even though the clonal evolution and the CSC models have been considered as mutually exclusive, both models could be complementary as intraclonal heterogeneity has been observed in tumors in which CSCs were identified [12].

The microenvironment within a tumor is not homogeneous. Differences in oxygen pressure, blood vessel density, growth factors, and composition of extracellular matrix are observed in human tumors. These differences affect tumor cells and might be the cause of phenotypic and genetic differences observed in tumor cells. Quintana *et al*. demonstrated that phenotypic heterogeneity in melanoma was largely driven by reversible changes in the expression of tumorigenic markers [7]. Tumor cell plasticity is a non-heritable source of heterogeneity that might explain some of the phenotypic differences between tumor cells and can be altered by the microenvironment [13] (Figure 1c). According to this view, the CSC model can be updated with the concept of various degrees of “stemness” and/or tumorigenic potential, determined either by microenvironmental cues or stochastic events [13,14]. This model would explain some incongruences of the CSC hypothesis as the ambiguous nature of the CSC, or the inconsistency of tumor transplantation depending on the experimental model used. Tumor cell plasticity might also be important in determining the drug resistance of tumor cells [15]. A more recent perspective, mostly based on results obtained with current state-of-the-art single-cell analyses, illustrates the clonal evolution of tumors as a growing tree (Figure 1d) [16,17]. The trunk represents the original clone, which carries mutations that will be shared by all cells in the tumor. Among these mutations, the ones that are both sufficient and necessary to initiate malignant transformation and to maintain tumor growth are defined as “driver” mutations. Other clones, originating by acquisition and accumulation of new mutations, are depicted as the branches that sprout from the trunk. Thus, the branches represent the genetic heterogeneity observed in tumors. Their heterogeneous mutations are mostly considered as “passenger” mutations, which in most cases are thought to confer a growth advantage only in the genetic background in which they originated. However, in some cases some of these alterations might become driver mutations, and ensure tumorigenic potential independent of the original driver events. 

The knowledge of the “tree structure” of a tumor would be predictive of the probability of treatment success: when branches are few a drug targeting a trunk mutation will have a good chance of promoting a positive outcome; on the contrary, when the branch structure is very complex (*i.e.*, in very heterogeneous tumors), it is likely that at least one clone will be able to overcome the effect of the therapy. 

The branched evolution model implicates and reinforces a previously proposed paradigm, known as interclonal cooperativity, which considers the functional interactions between different clones as key features to maintain heterogeneity and promote tumor growth [18,19]. According to this model, some clones possess a pro-oncogenic microenvironmental phenotype, which means the ability, through the production of extracellular factors, to modify the microenvironment to make it more favorable to the growth of other clones. These effects might include increase of proliferation rate, invasiveness, angiogenesis, immunosuppression, and metastasis. This kind of interclonal cooperativity has been demonstrated by the simultaneous engraftment of cells over-expressing wtEGFR or EGFRvIII, resulting in an actual acceleration of proliferation of the first, less tumorigenic cell line, or even in the acquisition of tumor forming ability in the case of Ink4/Arf null astrocytes [20].

## 3. Diagnostic and Therapeutic Implications of Heterogeneity

Tumor heterogeneity is defined by the presence of different cell populations or clones harboring distinct biologic, genetic or expression profiles within a tumor [13]. This tumor heterogeneity plays a crucial role in tumor growth, progression and resistance to therapy, but it can also confound diagnosis. 

Tumor sampling is key for diagnosis, as well as for treatment decisions. Conventionally, a single tumor piece is analyzed and used for diagnosis, however, this might be misleading since in the case of regional heterogeneity, a single sample would be lacking important information. These observations supported the development of “personalized” medicine where, using the information obtained from multiple samples, cocktails against several regionally dispersed drugable molecules can be designed. 

The co-existence of different clones can be explained when they have similar fitness, so that one subpopulation does not outgrow the other(s), but also when the group of subclones acts as a cooperative rather than competitive group for the more adapted clone. If the co-existence of different clones provides a survival benefit, targeting one of the key clones might be enough to collapse tumor growth by disrupting the interaction between the clones. Also, heterogeneity might be responsible for refractoriness to treatment due to pre-existence of clones resistant to the treatment or to interactions between clones that provide a protective environment against the treatment. Using sensitive methods, mutations conferring resistance to treatment, like the T790M mutation on EGFR, have been detected previous to treatment and a selection for resistant clones upon treatment was observed [2].

However, some authors suggest that mutations conferring resistance are acquired post-treatment, but these studies do not exclude the possibility of pre-existence of resistant cells in the tumor in a low proportion that would be difficult to detect depending on the sensibility of the method employed. For example, it has been reported that MSH6 gene mutations are not present in untreated GBM, but they are induced by temozolomide treatment causing resistance to the therapy [21,22]. 

Epigenetic inactivation of the O6-methylguanine-DNA methyltransferase (MGMT) gene in GBM is detected to be present with a heterogeneous pattern where MGMT expression is detected in distinct areas of positive tumor cells surrounded by negative cells [23] and its inactivation by promoter hypermethylation is associated with better response to alkylating agents such as temozolomide. 

Small-molecule inhibitors targeting individual RTKs have been used in several GBM clinical trials, however, little or no improvement in patient outcome was achieved. One of the reasons for RTK inhibitor monotherapy failure is the presence of other activated RTKs in the same tumor. Concomitant activation of various RTKs has been observed in human GBM [24]. It has been shown that targeting a single RTK is not effective because other RTKs can drive tumor growth and maintain the activation of downstream signaling, a phenomenon indicated as RTK-switching, making necessary the combination of drugs to reduce glioma cell survival [24]. Nonetheless, co-amplification of two RTKs in a single cell is rare and most cells within a heterogeneous tumor present amplification of only one RTK, suggesting that a specific RTK inhibitor will target only a subpopulation of tumor cells. This pattern of RTK amplification has been observed for PDGFRA and EGFR, and targeting either receptor is not sufficient to eliminate the tumor making necessary a combined treatment [25].

## 4. Techniques to Assess Heterogeneity

In the last few years, efforts have been made to study this extremely challenging aspect of tumor biology using state-of-the-art technologies. The first technical issue to assess heterogeneity both in the laboratory and upon cancer diagnosis is tumor sampling. Usually, analysis is made in one biopsy or small tumor sample that is processed and analyzed as a block or tumor homogenate. This approach has been used to identify the core pathways altered in GBM [26], as well as to identify molecular subgroups with putative prognostic with predictive value [27,28]. However, a recent study based on multiple tissue sampling demonstrated the presence of regions with characteristics of different subtypes within the same tumor [29]. This finding underlies the importance of an appropriate technique of tumor sampling for diagnostics, and illustrates how a single biopsy can be misleading at the time of decision for a specific treatment.

Similar approaches, based on taking small samples from different regions from a single tumor mass have been widely used to study heterogeneity [16,30]. Tumor multisampling has been found useful in cases of regional heterogeneity, which indeed occurs in some cancers, including GBM [31], however, studies performed using techniques able to detect specific alterations on single cells *in situ* have demonstrated the presence of alterations in intermixed cells. This is illustrated by fluorescent in situ hybridization (FISH), that is able to detect copy number alterations in a tumor section. Several studies have analyzed the amplification of EGFR, PDGFR, and MET in GBM, and demonstrated that these alterations can be mutually exclusive or co-existent in the same cell [25,32,33]. These studies demonstrated that in some cases genetically different cells are deeply intermingled and randomly sparse throughout the tumor mass, which makes necessary the use of sophisticated techniques capable of detecting specific alterations at the single cell level, since tumor homogenates analyzed by array CGH are not sensitive enough to detect alterations present in a limited number of cells [33]. However, even though multi-color FISH is more sensitive in detecting specific alterations at the single cell level, the number of alterations that can be detected in a single assay is limited and it is necessary to establish *a priori* the alterations to analyze. 

In the last few years, technological advances have increased the sensitivity of genome analysis techniques up to the single cell level. Thanks to whole genomic amplification and ultra deep sequencing it is possible now to detect mutations or amplification/deletion events in a single sorted cell. These techniques opened a new era in cancer research, not only because they provide an unprecedented deep picture of the complex genetics of cancers, but also because they give precious insight into the mechanisms of tumor clonal evolution.

Navin and colleagues reconstructed the phylogenetic tree of a heterogeneous breast cancer by clustering 100 single-sorted cells based on chromosome breakpoints. In this study they propose a model of “punctuated clonal evolution” characterized by sudden emergence of tumor clones in contrast with other models of gradual accumulation of mutations. They also identify a relatively abundant population of pseudodiploid cells which do not undergo clonal expansion implicated as the reservoir of genomic heterogeneity within the tumor [34].

The impressive amount of data generated by single cell deep sequencing techniques requires adequate mathematical tools. A recent study applied newly developed bioinformatic algorithms to reconstruct the genomic history of 21 breast cancers [35]. They analyzed the distribution of thousands of mutations and reconstructed the genetic identity of the ‘most-recent common ancestor’, the cell responsible for the generation of each cell in the tumor. This cell appeared surprisingly early in molecular time, and it is described as a long-lived quiescent cell that differentiates into several clones that can accumulate hundreds to thousands of mutations before the emergence of a dominant clone which undergoes the switch to a highly proliferative phenotype that initiates the formation of detectable tumor mass.

Some of these technologies that are based in single cell analysis have been applied to the study of GBM heterogeneity. Nickel and collaborators applied ultra-deep sequencing to detect mutations on a single DNA molecule rather than the combined signal from a mixture of cancer cells, with the goal to detect alterations present in a small minority of cells. They characterized tumor heterogeneity across tumor regions as well as over time in a GBM patient finding that some mutations were present only in a subset of cells within a region [3]. 

Another example of single-cell analysis in GBM is the employment of a newly described method based on a microfluidic platform capable to perform proteomic analysis at single-cell level. Sun *et al.* [36] developed and validated a microfluidic microscopy-based cytometry platform to characterize heterogeneity at single-cell level in primary brain tumor samples. Using this technology they quantified expression of four signaling proteins involved in GBM (EGFR, PTEN, phospho-Akt and phospho-S6) correlating their results with tumor progression and patient survival.

## 5. Heterogeneity Models

Human tumors derive from normal cells that throughout time accumulate genetic and epigenetic alterations. There are no established mutational events necessary for malignant transformation and it depends on the tumor cell type. Intra-tumor heterogeneity adds complexity in the study of cancer development and mathematical models have been developed to study the dynamics and evolution of this inherent tumor process. Iwasa *et al*. developed a stochastic mathematical model restricting the number of cells to a constant value over time where cells accumulate genetic and/or epigenetic alterations over generations [37]. However, this model is an over-simplification of what happens in a tumor where the number of cells is not limited and proliferation is uncontrolled. In a manuscript from the same group, Durrett *et al*. proposed a similar mathematical model but considering a population with exponential growth, where genetic drift and natural selection drive the progression and variability of tumors [38]. 

*In vivo* mouse models have also been useful to study heterogeneity. Genetically engineered glioma mouse models reproduce some of the histopathological features of human tumors depending on the original genetic mouse background [39]. In addition, some of these models accumulate additional alterations that are typically present in human gliomas extending the heterogeneity observed in these tumors. Even though these models are more suitable for the study of tumor-stroma interactions and preclinical testing of anticancer therapies, they are limited by the dependence on their original genetic background and because they do not account for the presence of genetically different cancer cells that can interact and might improve or impede the fitness of a heterogeneous population. By recapitulating heterogeneity in a fully controllable system based on engineered cell lines mixed in known proportions we were able to discover interaction between different clones that promoted the maintenance of the intra-tumor heterogeneity through the secretion of paracrine factors by mutant EGFR cells that promote the survival and proliferation of wild-type EGFR over-expressing cells [20]. 

Sottoriva and collaborators, by analyzing multiple regions from a GBM tumor, were able to construct a model of tumor progression in time and space [29]. They analyzed copy number alterations (CNA) in four to six samples per tumor in a cohort of 11 GBM patients. With the information obtained from this multisampling, they were able to reconstructed tumor phylogeny establishing a founder clone that contained the alterations common to all cell clones and reconstruct the evolution of the tumor by the accumulation of CNAs in different regions. 

## 6. Heterogeneity in GBM

GBM is the most malignant and frequent brain tumor in adults and is characterized by its marked intratumoral heterogeneity as the term multiforme indicates. It is also characterized by its proneness to infiltrate throughout the brain parenchyma, its robust angiogenesis and necrogenesis, as well, as its intense resistance to apoptosis and its genomic instability [24].

The first evidence of genetic heterogeneity in gliomas was demonstrated in freshly isolated cells from clinical specimens that presented markedly different karyotypes [40] and variable expression of antigenic markers [41]. Recently, extensive expression profiling studies have identified four subgroups in GBM: Mesenchymal, Classical, Neural, and Proneural, the latter subdivided in G-CIMP and non-G-CIMP. These insightful data for the first time put order to the inter-tumor heterogeneity of GBM, and gave hope to the possibility of a specific therapy based on tumor subtype [27,28,42]. 

Microdissection and comparative genomic hybridization analysis of different regions of single GBM tumor samples illustrated the presence of chromosomal aberrations common to the entire tumor, as well as, area-specific chromosomal alterations [43]. 

Other studies demonstrated the presence of specific alterations, such as p53 mutation in low grade gliomas or MGMT expression in GBM, only in a subset of tumor cells or in specific areas surrounded by negative cells [23,44]. 

One of the hallmarks of GBM is the amplification of the EGFR gene that is present in approximately 40%–60% of the GBM [24]. This amplification is commonly accompanied by a mutation in the same protein that gives rise to a constitutive active receptor that is known as EGFRvIII, ΔEGFR, or EGFRde2-7 [45]. Even though, EGFRvIII confers an enhanced tumorigenicity compared to the wild-type receptor, its expression is typically scattered and limited to a small subset of cells among wild-type positive cells [46]. A plausible explanation can be found in the observation comes that the EGFRvIII cells promote the survival and proliferation of wild-type EGFR cells through the paracrine secretion of soluble factors, such as IL-6 and LIF, or by generating a favorable microenvironment through the IL-8 mediated angiogenesis [20,47].

Heterogeneity in GBM has been observed either as regional or deeply intermixed heterogeneity. The first case has been described for the amplification of EGFR, which in some GBMs is found preferentially at the invading edge of the tumor, indicating a sort of “functional specialization” of a tumor subpopulation [33]. Little and collaborators observed regional distribution of PDGFRA and EGFR amplified clones, where PDGFRA amplified cells tended to be close to endothelial cells, while EGFR amplified cells were present in poorly vascularized regions [48]. On the contrary, in many reports, GBM cells create a mosaic of cells with different alterations. This latter pattern might be explained by the existence of interactions and cooperation between different clones, supported by experimental data [20], which indicates that some cells create a microenvironmental niche that sustains or favors the growth of other clones. 

Several groups have described a heterogeneous pattern of RTK amplification in GBM. The three most commonly amplified RTKs in GBM (EGFR, PDGFRA and MET) have been the object of these studies [25,33]. Dual-color FISH analysis revealed tumors with amplification of these three RTKs in a heterogeneous fashion, demonstrating that the vast majority of tumor cells presented amplification of only one RTK while fewer cells presented amplification of more than one RTK. Amplification of these RTKs have been observed in separated regions or intermingled throughout the tumors [25,32,33,48]. Snuderl *et al*. observed that in all GBMs analyzed, these clones presenting amplification of a unique RTK (PDGFRA or EGFR), shared a common genetic ancestor that was characterized by CDKN2A deletion or TP53 mutation [33]. Even though two RTKs are co-amplified in the same cell, transmission to the progeny is not assured and is observed at a high degree of mutual exclusivity if gene amplification occurs within adjacent tumor cells [48]. They proposed that maintenance of co-amplification is statistically unlikely if random segregation is assumed [25]. However, cell-to-cell interactions and selection might be considered since its influence might affect the proportion of co-amplified cells. 

Heterogeneity is probably found in every GBM, and its complexity has been found to correlate with the tumor degree [49,50]. Heterogeneous expression has also been described in GBM for angiopioetin-2 [51], MGMT [52], MMP-2 [53], and integrins [54]. In all cases these patterns are probably linked to the existence of specialized functional subdomains (angiogenic areas, invading front).

## 7. Conclusions

The deeper we look into tumor biology, the more evident it appears that a complete understanding of heterogeneity is mandatory in the fight against cancer. The advance of techniques, allowing detection of a single mutated DNA molecule, confirms and reinforces the concept hypothesized decades ago, that heterogeneity is a hallmark of several cancer types, in particular the most aggressive and incurable, GBM. These studies, which compiled genetic alterations in dozens to hundreds of cells throughout a tumor, not only illustrate how widespread heterogeneity is, but also show that a tumor is derived from a unique founder clone and that a number of alterations are shared by every cell in the tumor. This is likely the starting point of any personalized therapy. Attacking these mutations gives two advantages: first, to hit all the cells in the tumor, and second, to inhibit a process that is probably required for tumor maintenance. So will studying heterogeneity bring us back to simplicity? Probably it will not. We are learning that heterogeneity can affect tumor biology in a multiplicity of unexpected ways: by creating a chemoresistant microenvironment, or acquiring new driver mutations, or mutations that confer resistance, even the smallest represented clone can be responsible for therapy failure. The more complete a picture we can get of all mutations present in a tumor the more chances we will have to find the effective treatment. The technology to look into the genome of single cells is already a reality, and thanks to the continuous lowering of the costs, its applicability in diagnostics is becoming more and more feasible. Understanding how interactions between tumor clones might affect the effectiveness of a treatment and how to circumvent resistance will be the great challenge for the next decades.
